# Pharmacist-Led Continuous Glucose Monitoring for Prediabetes: Protocol for a Prospective Cohort Study

**DOI:** 10.2196/105275

**Published:** 2026-10-15

**Authors:** Kevin Cowart, Raechel T White, Nicholas W Carris, Benedicta O Ansong, Alyssa Hinden, Gwendolyn Clayton, John Petrilli, Eric Coris

**Affiliations:** 1 Department of Pharmacotherapeutics & Clinical Research University of South Florida Taneja College of Pharmacy Tampa, FL United States; 2 Department of Family Medicine University of South Florida Morsani College of Medicine Tampa, FL United States; 3 University of South Florida College of Public Health Tampa, FL United States

**Keywords:** prediabetes, primary care, continuous glucose monitoring, pharmacist, health behavior, diabetes prevention

## Abstract

**Background:**

Prediabetes represents a critical intervention window to reduce progression to type 2 diabetes. The current standard of care for treatment of prediabetes is weight loss and lifestyle modification, which are both highly effective, although limited by real-world adherence. It is well recognized that continuous glucose monitoring (CGM) improves glycemic and behavioral outcomes in people with diabetes, although outcomes among people with prediabetes are not well described.

**Objective:**

The aim of this study is to evaluate the impact of pharmacist-led CGM on glycemic control and health-related behaviors in adults with prediabetes.

**Methods:**

This study includes a prospective pharmacist-led CGM cohort and a matched retrospective usual-care cohort. Adults aged 18 to 70 years with prediabetes (hemoglobin A_1c_ [HbA_1c_] 5.7%-6.4%) will be eligible for the prospective cohort if they have a compatible smartphone and no CGM use in the prior 6 months. Participants will receive CGM (Stelo glucose biosensor; Dexcom) and diabetes self-management education and support over 12 weeks. The primary outcome is the change in HbA_1c_ at 12 weeks, compared between groups. Secondary outcomes include CGM-derived metrics, changes in Summary of Diabetes Self-Care Activities scores and 36-Item Short Form Health Survey scores, and CGM-related adverse effects.

**Results:**

This study was funded in 2026. Recruitment is ongoing, and no participants had been enrolled at the time of writing. The planned sample size is 40 participants: 20 prospective CGM participants and 20 retrospective controls. Outcome data are not yet available. The study is expected to be completed in July 2027, and the results are anticipated to be disseminated in December 2027.

**Conclusions:**

This study seeks to evaluate the glycemic impact, behavior change, and safety of pharmacist-led CGM for prediabetes in primary care.

**Trial Registration:**

ClinicalTrials.gov NCT07529366; https://tinyurl.com/33bv7u8c

**International Registered Report Identifier (IRRID):**

PRR1-10.2196/105275

## Introduction

Prediabetes is an important target for diabetes prevention [1-3]. More than 1 in 3 adults in the United States has prediabetes [4]. Evidence-based lifestyle modification remains the cornerstone of prevention [5]. However, long-term adherence to dietary change, physical activity, and weight-management behaviors is difficult to sustain [6-10]. Traditional counseling may be perceived as abstract because patients often do not receive immediate physiological feedback showing how daily behaviors influence glycemia [11].

Continuous glucose monitoring (CGM) may help address this gap by translating food choices, physical activity, sleep patterns, and other behaviors into personalized glucose trends that people with prediabetes can view in real time [11-13]. In diabetes care, CGM is increasingly used to guide therapy and support self-management [14]. However, its role in prediabetes is less established, particularly as a preventive feedback tool delivered within primary care rather than as a device-only intervention. Over-the-counter CGM systems have made glucose sensing more accessible to adults who do not use insulin, creating a practical opportunity to evaluate CGM as a behavioral intervention for people with prediabetes.

Pharmacists are well positioned to deliver this intervention in primary care. Prior pharmacist-led CGM and diabetes care models suggest potential for improved glycemic outcomes, patient engagement, and satisfaction [15-19]. Pharmacists positioned in primary care routinely provide chronic disease medication management, order laboratory tests, and recommend or prescribe medications in collaboration with medical providers [20-22]. Pairing CGM with pharmacist-led education and structured diabetes self-management education and support (DSMES) may allow patients to link glucose data with modifiable behaviors and may allow clinicians to reinforce individualized diabetes prevention strategies.

## Methods

### Study Objectives

The primary objective of this study is to determine the impact of pharmacist-led CGM on glycemic control in people with prediabetes. The secondary objectives are to assess changes in CGM-derived glycemic outcomes, health-related behaviors, and safety outcomes.

### Practice Setting

The study will be conducted in the University of South Florida (USF) Health Department of Family Medicine, where clinical pharmacists provide chronic disease state management under a protocol in collaboration with medical providers. The use of CGM data review, glucose education, laboratory monitoring, and medication review is integrated into routine pharmacist appointments. The research components include the provision of study-funded CGM sensors and the systematic collection and analysis of glycemic, behavioral, and safety outcomes.

### Study Design

This is a single-site study using a prospective cohort design with a matched retrospective chart review control group. The prospective intervention cohort will receive pharmacist-led CGM and DSMES over 12 weeks. The inclusion and exclusion criteria are presented in Textbox 1. Enrolled participants in the intervention group will wear a CGM sensor (Stelo glucose biosensor; n=20). Each participant in the intervention group will have 4 visits with a pharmacist. The retrospective control group (n=20) will comprise adults with prediabetes who received usual care without pharmacist-led CGM during the historical study period (January 1, 2025-March 20, 2026). This study was registered with ClinicalTrials.gov (NCT07529366) on April 7, 2026.

**Inclusion and exclusion criteria.**

**Inclusion criteria**

- Adults aged 18-70 years
- Prediabetes (hemoglobin A_1c_ (HbA_1c_): 5.7%-6.4%)
- A compatible smartphone with the Dexcom Stelo glucose biosensor
- Have not worn a continuous glucose monitoring (CGM) device in the 6 months prior to enrollment

**Exclusion criteria**

- Any of the following forms of diabetes: type 1 diabetes, type 2 diabetes, monogenic diabetes, cystic fibrosis–related diabetes, posttransplant diabetes, and latent autoimmune diabetes
- Problematic hypoglycemia in the prior 6 months (defined as recurrent [more than 1] level 2 hypoglycemic events [glucose <54 mg/dL] that persist despite multiple attempts to adjust medications and/or modify the diabetes or treatment plan, or a history of 1 level 3 hypoglycemic event [glucose <54 mg/dL] characterized by an altered mental and/or physical state requiring third-party assistance for treatment of hypoglycemia)
- Pregnancy or plans to become pregnant during the study time frame
- Current or planned dialysis during the study time frame, or current use of systemic steroids for any condition
- A known allergy to medical-grade adhesives
- Use of any CGM device in the past 6 months
- A history of a diagnosed eating disorder
- Current use of a second-generation antipsychotic at the time of consent (aripiprazole, asenapine, brexpiprazole, cariprazine, clozapine, iloperidone, lurasidone, olanzapine, paliperidone, quetiapine, risperidone, or ziprasidone)
- Participant lacks the capacity to provide informed consent

### Ethical Considerations

This study was approved by the USF Institutional Review Board (IRB; STUDY009771). All participants will provide informed consent before enrollment after discussion with a member of the study team. All personal information and study data collected will be stored in REDCap (Vanderbilt university), a secure, Health Insurance Portability and Accountability Act (HIPAA)–compliant web application hosted on encrypted USF servers. All research activities will be conducted in accordance with the Declaration of Helsinki. All data will be deleted 5 years after the study has concluded. Confidential data will not be disclosed outside the study team except as required by law or as permitted by the informed consent (eg, with the USF IRB or those who monitor the study). All data will be deidentified for the analysis.

### Recruitment

Prospective participants will be identified through (1) routine pharmacist care that includes referrals from medical providers within the department for comanagement of prediabetes, (2) identification of participants through quarterly reports of patients seen in the USF Health Department of Family Medicine during the previous 12 months, and (3) prospective pharmacist review of clinic schedules within the department. Potentially eligible patients may be contacted on behalf of their primary care provider through the electronic health record (EHR) patient portal, by telephone, or during a clinic visit, after provider agreement. A pharmacist will conduct a brief prescreening to confirm eligibility, including smartphone compatibility and no CGM use in the prior 6 months. Written informed consent will be obtained before any study-specific procedures.

The control group will be identified retrospectively from the EHR. The index date will be defined as the first medical provider visit during the control group study time frame, January 1, 2025, to March 20, 2026. Controls will be adults aged 18 to 70 years with prediabetes, no documented CGM use within the prior 6 months, and available baseline and eligible 12-week follow-up HbA_1c_ values.

### Sample Size

This study is designed with a planned sample of 40 participants: 20 prospective participants receiving pharmacist-led CGM and 20 matched historical controls. Because no study has evaluated the identical intervention, the power assumptions were taken from existing literature in people with prediabetes in which CGM was evaluated or similar interventions were tested [11,23-27]. A conservative 2-sample *t* test was used because the baseline–follow-up correlation and within-pair correlation needed to quantify the efficiency gain from the planned analysis of covariance and matched analysis were unavailable. With 20 participants per group, a 2-sided α of .05, an expected difference of 0.2%, and an SD of 0.3%, the planned sample provides 47% power. The calculation assumes no missing primary outcome data. Retrospective controls will be eligible for matching only if both baseline and eligible follow-up HbA_1c_ values are available. Therefore, these results do not provide a definitive test of intervention efficacy. Our objectives are to assess feasibility and estimate the magnitude and variability of the intervention effect size to inform a future adequately powered study. Under these assumptions, a future prospectively randomized study would require approximately 44 participants per group, or 88 participants overall, to provide 80% power to detect a 0.2% between-group difference using a 2-sided α of .05.

### Study Visits

An outline of activities occurring at each study visit is shown in Table 1. At the baseline visit, eligibility and contraindications will be confirmed, informed consent will be obtained, demographics and medical history will be collected, vital signs will be recorded (blood pressure, pulse, body weight, and BMI), medications will be reviewed, diet and physical activity will be assessed, and baseline HbA_1c_ will be measured from a fingerstick blood sample. Participants will complete the Summary of Diabetes Self-Care Activities (SDSCA) questionnaire [28] and the 36-Item Short Form Health Survey (SF-36) [29]. Participants will have a CGM sensor placed and will receive standardized education on CGM use and interpretation, as well as DSMES focused on practical application of glucose data to diet, physical activity, and daily routines. The core DSMES content to be provided is evidence based and in line with the most recent national standards and focuses on pathophysiology, healthy coping, healthy eating, being active, taking medication, monitoring, reducing risk, problem-solving, and behavior change strategies [30].

**Table 1:** Study-related activities at each visit.

Study activity	Visit 1 (baseline)	Visit 2 (wk 2)	Visit 3 (wk 4)	Visit 4 (wk 12)
Obtain informed consent	✓			
Confirm eligibility or ensure no contraindications	✓			
Collect baseline characteristics and past medical history	✓			
Collect vital signs^a^	✓	✓	✓	✓
Assess medications	✓	✓	✓	✓
Assess diet and physical activity	✓	✓	✓	✓
Collect HbA_1c_^b^ blood sample	✓			✓
CGM^c^ sensor placement and education	✓	✓		
Assess for CGM sensor adverse effects		✓	✓	
Review the glucose profile and upload it to the EHR^d^		✓	✓	
Administer structured DSMES^e^	✓	✓	✓	✓
Administer SDSCA^f^	✓		✓	✓
Administer SF-36^g^	✓		✓	✓
Visit type	Clinic	Clinic or telehealth	Clinic or telehealth	Clinic

^a^Collected during clinic visits.

^b^HbA_1c_: hemoglobin A_1c_.

^c^CGM: continuous glucose monitoring.

^d^EHR: electronic health record.

^e^DSMES: diabetes self-management education and support.

^f^SDSCA: Summary of Diabetes Self-Care Activities.

^g^SF-36: 36-Item Short Form Health Survey.

At week 2, vital signs, medications, diet, and physical activity will be reassessed; CGM use and adverse effects will be reviewed; glucose data will be uploaded to the EHR; and DSMES will be reinforced. A new CGM sensor will be applied at the week 2 visit. Medication changes, if clinically indicated, will be handled as routine care under the existing clinic protocol and practice model and will not be research interventions. At week 4, the same clinical review will be conducted, with repeat administration of the SDSCA and SF-36. At week 12, HbA_1c_ will be repeated, vital signs and medications will be reassessed, DSMES will be reinforced, and the final SDSCA and SF-36 will be completed. Missed visits will be rescheduled within approximately 1 to 2 weeks, when possible.

### Comparator

The comparator is a matched historical usual-care cohort also receiving care in the USF Health Department of Family Medicine without pharmacist-led CGM. Retrospective controls will be matched to prospective participants on baseline HbA_1c_, age, gender, and BMI, where feasible. Additional matching or adjustment variables may include baseline weight and diabetes-related or weight-loss medications, if applicable.

### Outcomes

The primary outcome is the change in HbA_1c_ at 12 weeks, compared between the pharmacist-led CGM cohort and matched historical controls. For controls, baseline HbA_1c_ will be obtained at the index visit during the study period, and the follow-up HbA_1c_ will be the value closest to 12 weeks after the index visit within the study period.

As shown in Table 2, secondary glycemic outcomes in the prospective cohort include within-cohort changes in CGM-derived glycemic metrics from baseline to the end of the 4-week CGM wear period. These include the number and percentage of participants achieving various CGM metrics, including time in range, mean sensor glucose concentration, time below range, time above range, glycemic variability, a 5% and 10% increase in time in range, and a change in the number of participants achieving more than 70% time in range.

**Table 2:** Secondary glycemic outcomes.

Outcome measure	Measure description	Time frame
Time in range	Change in the time in range (70-140 mg) at 4 weeks, %, and compared within the intervention cohort	Baseline to end of week 4 (CGM^a^ wear period)
Mean sensor glucose	Change in mean sensor glucose concentration at 4 weeks, mg/dL, and compared within the intervention cohort	Baseline to end of week 4 (CGM wear period)
Time below range	Change in time below range (<70 mg/dL) at 4 weeks, %, and compared within the intervention cohort	Baseline to end of week 4 (CGM wear period)
Time above range	Change in time above range (141-250 mg/dL and >250 mg/dL) at 4 weeks, %, and compared within the intervention cohort	Baseline to end of week 4 (CGM wear period)
Glycemic variability	Change in glycemic variability (SD and coefficient of variance) at 4 weeks and compared within the intervention cohort	Baseline to end of week 4 (CGM wear period)
Time in range increase of 5%	Change in the number of participants with a ≥5% increase in time in range (70-140 mg/dL) at 4 weeks and compared within the intervention cohort	Baseline to end of week 4 (CGM wear period)
Time in range increase of 10%	Change in the number of participants with a ≥10% increase in time in range (70-140 mg/dL) at 4 weeks and compared within the intervention cohort	Baseline to end of week 4 (CGM wear period)
Participants achieving >70% time in range	Change in the number of participants achieving >70% time in range (70-140 mg/dL) at 4 weeks and compared within the intervention cohort	Baseline to end of week 4 (CGM wear period)

^a^CGM: continuous glucose monitoring.

Additional secondary outcomes include changes in diabetes self-care behaviors and health-related quality of life, assessed using the SDSCA and SF-36. Safety end points will include assessment of CGM sensor adverse effects, including cutaneous reactions at the site where the CGM sensor is placed. Specific sensor site reactions that will be assessed include erythema, itching, rash, pain, bleeding, bruising, edema, infection, and induration. If any participant experiences a serious cutaneous reaction to the sensor, defined as infection, moderate or severe pain (a score of 4 or greater out of 10), or serious bleeding, they will be withdrawn from the study. If 3 participants are withdrawn for this reason, the study will be stopped.

### Statistical Methods

Baseline characteristics will be summarized using descriptive statistics. Continuous variables will be reported as mean (SD) or median (IQR), as appropriate; categorical variables will be reported as counts and percentages.

The primary analysis will use analysis of covariance with HbA_1c_ at 12 weeks as the dependent variable, study group as the main predictor, baseline HbA_1c_ as a covariate, and indicator variables for matched-pair membership (matched-pair fixed effects). This model estimates the group effect within matched pairs while accounting for residual within-pair differences in baseline HbA_1c_. Because of the pilot sample size, no additional covariates will be included in the primary model. The study-group coefficient will be reported as the adjusted mean between-group difference in HbA_1c_ at 12 weeks, expressed in percentage points, with a 95% CI and a 2-sided *P* value, with α set at .05. If any HbA_1c_ values are missing at 12 weeks, contrary to the design assumption, the number, group, and reasons will be described.

Within the prospective cohort, changes in CGM-derived metrics will be summarized descriptively. Repeated continuous outcomes may be analyzed using linear mixed-effects models with participant-level random effects. Binary outcomes may be analyzed using generalized linear mixed models with a logit link. Changes in SDSCA and SF-36 scores will be analyzed using paired *t* tests or their nonparametric equivalents, depending on the distributional assumptions.

### Dissemination Plan

The findings of this prospective cohort study will be disseminated through publication in national and international peer-reviewed scientific journals and presentation at annual professional conferences.

## Results

This study will enroll 20 adults with prediabetes into the prospective pharmacist-led CGM cohort and will identify 20 matched retrospective controls from patients seen between January 1, 2025, and March 20, 2026. At the time of manuscript preparation, no participants had been enrolled, and outcomes were not available.

## Discussion

### Anticipated Findings

Prediabetes represents one of the largest and most actionable populations for diabetes prevention [31,32]. An estimated 115.2 million adults in the United States have prediabetes; however, approximately 8 in 10 are unaware of their condition, underscoring a major gap in risk recognition with significant public health implications [33]. Because progression from prediabetes to type 2 diabetes is preventable, this period represents a critical window for intervention [1]. CGM may be uniquely suited for prediabetes because it translates an otherwise silent risk state into visible, individualized feedback. This study therefore addresses a clinically relevant and underexplored question for primary care: whether short-term visualization of glucose patterns can motivate lifestyle change and improve glycemic measures.

Evidence suggests that CGM combined with lifestyle or dietary coaching is feasible and acceptable among adults with prediabetes [11,34]. However, most pharmacist-led CGM literature has focused on adults with established diabetes, in whom CGM data have been used to support patient education, interpret glucose patterns, guide pharmacological interventions, and coordinate diabetes management [35,36]. Pharmacist-led CGM has been associated with improvements in HbA_1c_ and time in range [35,36]. However, evidence has largely emphasized glycemic outcomes rather than behavioral, humanistic, or prevention-focused outcomes. Thus, the novelty of this study lies in adapting a pharmacist-led CGM care model from diabetes management to diabetes prevention, pairing short-term CGM visualization with DSMES to assess whether pharmacists can implement a CGM intervention providing individualized glucose feedback to improve risk awareness, reinforce lifestyle change, and affect early glycemic measures among adults with prediabetes.

### Strengths and Limitations

Strengths include the primary care setting, structured DSMES, repeated patient-reported measures, and the assessment of both clinical and behavioral outcomes. The design is intended to reflect a practical clinical workflow that occurs in the real world, is generalizable, and can be scaled. The matched retrospective comparator is intended to provide a feasible usual-care comparison while minimizing contamination that could occur if patients in the same clinic were concurrently randomized to receive or not receive pharmacist-led CGM.

Limitations include the single-site sample, relatively short 12-week follow-up, nonrandomized design, and reliance on data from historical controls. Matching and adjusted analyses will mitigate, but cannot eliminate, confounding. Additionally, smartphone compatibility requirements may exclude some patients and could possibly limit generalizability to a younger population more comfortable with diabetes technology. Furthermore, CGM use may itself prompt increased attention to diet and activity during the wear period; however, the durability of behavior change following CGM sensor use can only be established through a long-term clinical trial. Requiring retrospective controls to have HbA_1c_ measurements at baseline and 12 weeks may introduce selection bias toward patients who received more frequent laboratory monitoring or health care. If there are insufficient retrospective controls with complete HbA_1c_ data, given the timing and frequency of routine HbA_1c_ monitoring in real-world practice, the HbA_1c_ value closest to the 12-week time point will be used.

### Conclusions

This study will evaluate pharmacist-led CGM as a technology-enabled behavioral intervention for adults with prediabetes in primary care. By combining pharmacist-led CGM and structured DSMES, the study will generate evidence on glycemic impact, self-care behaviors, quality of life, and safety.

## Appendix

Multimedia Appendix 1 Peer-reviewer report from the ASHP (American Society of Health-related Pharmacists).

## Abbreviations

CGM: continuous glucose monitoring
DSMES: diabetes self-management education and support
EHR: electronic health record
HbA_1c_: hemoglobin A_1c_
HIPAA: Health Insurance Portability and Accountability Act
IRB: institutional review board
SDSCA: Summary of Diabetes Self-Care Activities
SF-36: 36-Item Short Form Health Survey
USF: University of South Florida

## Data Availability

No study data are included in this protocol. The datasets generated or analyzed during this study will not be made publicly available because the institutional review board does not permit their deposit in a public repository.
