# Supplementary material for: Pharmacist-Led Continuous Glucose Monitoring for Prediabetes: Protocol for a Prospective Cohort Study
Source: JMIR Res Protoc. 2026 Oct 15;15:e105275. doi: 10.2196/105275 (PMC13720402; doi:10.2196/105275)
Supplement: Supplementary file 1 [file resprot_v15i1e105275_app1.pdf]

Submission Review Report

**Application**	2025 Pharmacy Resident Practice-Based Research Grant
**Submission Title**	Redefining Prediabetes Care: Pharmacist-Led Continuous Glucose Monitoring to Drive Lifestyle Change
**Institution**	University of South Florida Taneja College of Pharmacy
**Total Reviews**	5

At-a-Glance

Total Score	Overall Impact	Reviews
**94.50 / 100**	**1.75**	**5**
Adjusted mean score	Scale: 1.0 - 9.0	Total reviews received

Criteria Scoring

Criterion	Points Possible	Raw Mean	Adjusted Mean	Range
Specific Aims and Hypothesis	20.0	19.75	19.75	19 - 20
Rationale and Significance	10.0	9.75	9.75	9 - 10
Innovation	10.0	10.00	10.00	10 - 10
Investigators and Environment	15.0	14.25	14.25	12 - 15
Approach	40.0	36.00	36.00	30 - 40
Scope and Timeline	5.0	4.75	4.75	4 - 5
**Total**	100	94.50	94.50	
**Overall Impact Scale**	1.0 - 9.0	1.75	1.75	1 - 3

Full Reviewer Comments by Criterion

Specific Aims and Hypothesis

**Reviewer 2**	Very well written, and I appreciate the clear aims with related hypotheses.

**Reviewer 3**	The specific aims are exceptionally clear, well justified, and logically sequenced. Each aim is paired with a well-crafted hypothesis tied to measurable outcomes (CGM-derived metrics, A1c, and behavioral changes). The structure aligns tightly with the clinical relevance and innovation of applying CGM in prediabetes. One minor improvement would be specifying your predefined clinical thresholds or effect sizes where appropriate, but overall the aims are outstanding.

**Reviewer 4**	Patient access, technology solution, and patient-centered care are all research priorities. Aims are straightforward, and the use of TIR/TAR to make the analysis patient-specific is a great option.

**Reviewer 5**	Very clear aims with working hypotheses for each. They align well with ASHP recommendations toward integrated informatics and innovation within the pharmacy paradigm.

Rationale and Significance

**Reviewer 3**	This is an extremely compelling rationale. The proposal articulates the major public health burden of prediabetes and effectively frames the lack of real-world evidence for CGM use in this population as a critical gap. You provide strong justification for pharmacist leadership in this work, supported by robust citations and preliminary institutional experience. The narrative thoroughly addresses significance at the patient, population, and system levels. This section is exemplary.

**Reviewer 4**	Well referenced. Goes beyond filling a gap - practicing health care versus sick care, which is an approach our health care system does not do enough of.

Innovation

**Reviewer 2**	Very innovative and cool pilot project!

**Reviewer 3**	This is one of the most innovative projects submitted this cycle. The proposal clearly explains how the intervention challenges the traditional model of glycemic monitoring, positioning CGM as a behavioral feedback tool rather than a medication-titration instrument. The novelty is well substantiated: pharmacist-led CGM in prediabetes is largely unexplored, and embedding technology-enabled behavioral coaching into primary care represents a meaningful expansion of the pharmacist role. This section articulates a forward-looking, paradigm-shifting approach.

**Reviewer 4**	Agree with the author - prediabetes work is nascent, and the real-time feedback opportunity may drive improved results.

Investigators and Environment

**Reviewer 4**	All bases of practice are covered for expertise: ambulatory care experience, diabetes researcher experience, experience in pharmacist-led interventions, and a biostatistician who has prediabetes expertise.

**Reviewer 5**	Recommend integrating an endocrinologist into the research team, if possible. This will facilitate a complete, 360-degree view and a multidisciplinary model.

Approach

**Reviewer 2**	Very detailed and thorough approach! My only concern is that you have planned a very detailed statistical analysis for a total cohort of only 40 people. Methods like propensity matching and regression analyses are not usually appropriate for sample sizes this small. The descriptive statistics should be enough for this pilot project!

**Reviewer 4**	Inclusion/exclusion criteria are appropriate, and the sampled data appear to support the availability of the population, although needing 90 days of wear data makes this challenging to confirm. The proposal uses study software to manage patient data and includes a plan for discontinuation if patients experience issues. The analysis plan is clearly outlined and includes prespecified subgroup analyses. I appreciate Tables 2 and 3 showing the plan for each visit and the metrics. The proposal states exactly what A1c tests are utilized and plans to match patients from the retrospective chart review with propensity score matching to minimize confounding. Overall, the study plan is very well thought out.

**Reviewer 5**	The study relies on weekly visits. What is the plan if appointments are rescheduled or if patients are lost to follow-up? Will the enrolled patient cohort of 20 continue to grow until 20 complete patients are included for all data points?

General

**Reviewer 3**	This is an exceptionally high-quality resident research proposal. The writing is polished and cohesive, the methodology is rigorous, and the project directly addresses a national gap in diabetes prevention research. The intervention is innovative, clinically relevant, and scalable. This proposal is likely to produce publishable results and elevate the role of pharmacist-led CGM services across primary care settings.

**Reviewer 5**	Outstanding proposal with clear aims, methodologies, and plans for statistical analysis. This aligns with the ASHP PAI - I look forward to reading the publication.
